# [(1,2,5,6-η)-Cyclo­octa-1,5-diene]bis­(4-methyl­phen­yl)platinum(II)

**DOI:** 10.1107/S1600536810049664

**Published:** 2010-12-04

**Authors:** Zhi-Wei Wang, Ran Liu, Hong-Yu Liu, Chong-Qing Wan

**Affiliations:** a Department of Chemistry, Capital Normal University, Beijing 100048, People’s Republic of China

## Abstract

In the mononuclear title complex, [Pt(C_7_H_7_)_2_(C_8_H_12_)], the Pt^II^ ion exhibits a square-planar coordination geometry defined by two methyl­phenyl ligands and the mid-points of the two π-coordinated double bonds of cyclo­octa-1,5-diene. The two methyl­phenyl groups have a *cis* relationship with a C—Pt—C bond angle of 88.54 (18)° and a dihedral angle between the mean planes of the benzene rings of 83.87 (1)°. Each complex mol­ecule links to four symmetry-related ones through inter­molecular C—H⋯π inter­actions, forming a layer almost parallel to the *bc* plane.

## Related literature

For general background to Pt^II^ complexes with cyclo­octa-1,5-diene, see: Goel *et al.* (1982) ▶; Syed *et al.* (1984 ▶). For the structures of analogous Pt^II^ complexes, see: Deacon *et al.* (1993 ▶); Debaerdemaeker *et al.* (1987 ▶, 1991 ▶); Roviello *et al.* (2006 ▶). For C—H⋯π inter­actions, see: Umezawa *et al.* (1998 ▶). For the preparation, see: Chaudhury & Puddephatt (1975 ▶).
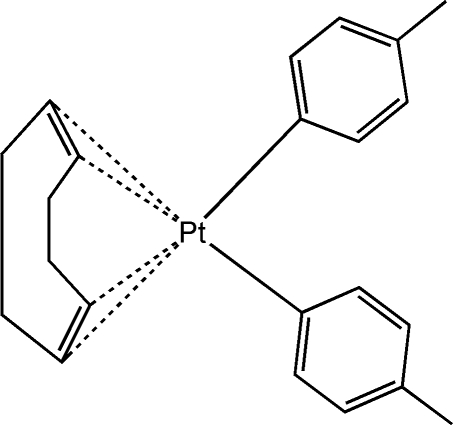


## Experimental

### 

#### Crystal data

[Pt(C_7_H_7_)_2_(C_8_H_12_)]
*M* *_r_* = 485.52
Monoclinic, 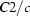

*a* = 25.029 (13) Å
*b* = 8.172 (4) Å
*c* = 19.674 (10) Å
β = 118.417 (8)°
*V* = 3539 (3) Å^3^
*Z* = 8
Mo *K*α radiation
μ = 7.93 mm^−1^
*T* = 293 K
0.36 × 0.30 × 0.20 mm

#### Data collection

Bruker APEXII CCD area-detector diffractometer
Absorption correction: multi-scan (*SADABS*; Bruker, 2007 ▶) *T*_min_ = 0.584, *T*_max_ = 1.000
8906 measured reflections
3113 independent reflections
2884 reflections with *I* > 2σ(*I*)
*R*_int_ = 0.023

#### Refinement

*R*[*F* ^2^ > 2σ(*F* ^2^)] = 0.022
*wR*(*F* ^2^) = 0.061
*S* = 1.11
3113 reflections
208 parameters
H-atom parameters constrained
Δρ_max_ = 2.74 e Å^−3^
Δρ_min_ = −0.99 e Å^−3^

### 

Data collection: *APEX2* (Bruker, 2007 ▶); cell refinement: *APEX2* and *SAINT* (Bruker, 2007 ▶); data reduction: *SAINT*; program(s) used to solve structure: *SHELXS97* (Sheldrick, 2008 ▶); program(s) used to refine structure: *SHELXL97* (Sheldrick, 2008 ▶); molecular graphics: *SHELXTL* (Sheldrick, 2008 ▶); software used to prepare material for publication: *SHELXTL* and *PLATON* (Spek, 2009 ▶).

## Supplementary Material

Crystal structure: contains datablocks I, global. DOI: 10.1107/S1600536810049664/zq2077sup1.cif

Structure factors: contains datablocks I. DOI: 10.1107/S1600536810049664/zq2077Isup2.hkl

Additional supplementary materials:  crystallographic information; 3D view; checkCIF report

## Figures and Tables

**Table 1:** Hydrogen-bond geometry (Å, °) *Cg*1 and *Cg*2 are the centroids of the C9–C14 and C2–C7 rings, respectively.

*D*—H⋯*A*	*D*—H	H⋯*A*	*D*⋯*A*	*D*—H⋯*A*
C1—H1*B*⋯*Cg*1^i^	0.96	2.93	3.615 (4)	129
C20—H20*B*⋯*Cg*1^ii^	0.96	2.85	3.749 (5)	155
C21—H21*A*⋯*Cg*2^ii^	0.97	2.83	3.411 (4)	119
C8—H8*C*⋯*Cg*2^iii^	0.96	2.85	3.509 (2)	126

Symmetry codes: (i) 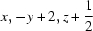; (ii) 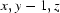; (iii) 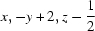.

**Fig. 1.**
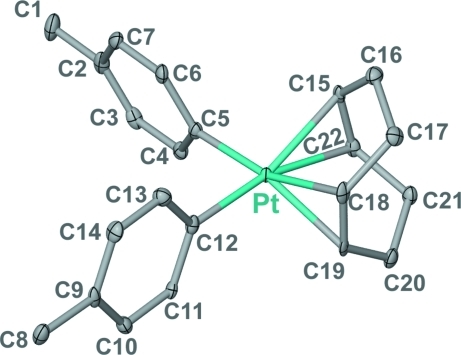
The atom-numbering scheme of the title complex. Displacement ellipsoids are drawn at the 30% probability level and H atoms are omitted for clarity.

**Fig. 2.**
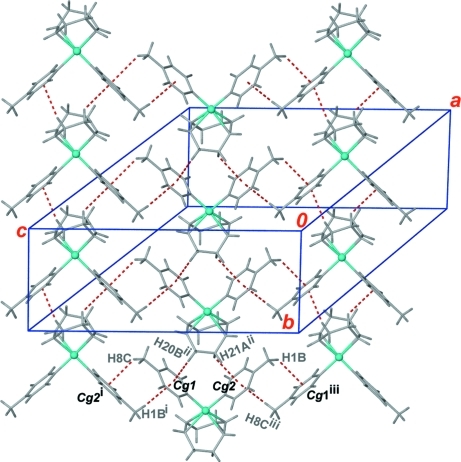
The C—H(methyl)···π and C—H(methylene)···π interactions between the mononuclear units, forming a layer in the bc plane. The Cg1 and Cg2 are the centroids of the C9—C10—C11—C12—C13—C14 and C2—C3—C4—C5—C6—C7 rings, respectively. Symmetry codes: (i) x, -y + 2, z - 1/2; (ii) x, y + 1, z; (iii) x, -y + 2, z + 1/2.

## supplementary crystallographic information

### Comment

The Pt^II^ complexes with cycloocta-1,5-diene (COD) are versatile precursors in inorganic synthesis (Goel *et al.*, 1982; Syed *et al.*, 1984). Herein, we report the structure of the bis-aryl complex [(COD)Pt(C_7_H_7_)_2_]. In the crystal structure of the title complex, the center Pt^II^ adopts a square-planer coordination geometry with two methylphenyl groups depositing in a *cis* relationship, and the cycloocta-1,5-diene bonding to the ion with a 1,2,5,6-η^4^-coordination mode (Fig. 1). The Pt1—C5 and Pt1—C12 bond lengths equal 2.028 (4) Å, while the distances from the Pt^II^ to the doubly-bonded C atoms lie within the range of 2.256 (4)–2.279 (4) Å, all of which are comparable to that of similar complexes. The two methylphenyl groups site in a *cis* relationship with a C5—Pt1—C12 bond angle of 88.54 (18)° and a dihedral angle between the two benzene rings of 83.87 (1)°. Each of such mononuclear complex moiety links four symmetry-related ones through two types of intermolecular C—H···π interactions [C—H(methylene)···π and C—H(methyl)···π] to form a layer almost parallel to the *bc* plane, as shown in Fig. 2. The C···centroid distances vary from 3.411 (4) to 3.749 (5) Å, and C—H···centroid bond angles lie within the range of 119–155° (Umezawa *et al.* 1998).

### Experimental

The title complex was obtained following a reaction procedure from literature (Chaudhury *et al.*, 1975). Reaction of aryl Grignard reagents (C_6_H_4_-4-CH_3_)MgBr (0.195 g, 1 mmol) with (COD)PtCl_2_ (0.086 g, 0.8 mmol) in ether formed the title compound as a white powder, crystals of which were obtained after four days by recrystallization from CH_2_Cl_2_/n-hexane, yield: 0.233 g (60%).

### Refinement

The hydrogen atoms were placed in idealized positions and allowed to ride on the relevant carbon atoms, with C—H = 0.93 and 0.97 Å for aryl and methylene H atoms, respectively, and with *U_iso_*(H) = 1.2*U_eq_*(C).

### Crystal data

**Table:**

[Pt(C_7_H_7_)_2_(C_8_H_12_)]	*Z* = 8
*M**_r_* = 485.52	*F*(000) = 1888
Monoclinic, *C*2/*c*	*D*_x_ = 1.823 Mg m^−^^3^
Hall symbol: -C 2yc	Mo *K*α radiation, λ = 0.71073 Å
*a* = 25.029 (13) Å	µ = 7.93 mm^−^^1^
*b* = 8.172 (4) Å	*T* = 293 K
*c* = 19.674 (10) Å	Block, colourless
β = 118.417 (8)°	0.36 × 0.30 × 0.20 mm
*V* = 3539 (3) Å^3^	

### Data collection

**Table:**

Bruker APEXII CCD area-detector diffractometer	3113 independent reflections
Radiation source: fine-focus sealed tube	2884 reflections with *I* > 2σ(*I*)
graphite	*R*_int_ = 0.023
ω scans	θ_max_ = 25.0°, θ_min_ = 2.2°
Absorption correction: multi-scan (*SADABS*; Bruker, 2007)	*h* = −29→17
*T*_min_ = 0.584, *T*_max_ = 1.000	*k* = −9→9
8906 measured reflections	*l* = −20→23

### Refinement

**Table:**

Refinement on *F*^2^	Primary atom site location: structure-invariant direct methods
Least-squares matrix: full	Secondary atom site location: difference Fourier map
*R*[*F*^2^ > 2σ(*F*^2^)] = 0.022	Hydrogen site location: inferred from neighbouring sites
*wR*(*F*^2^) = 0.061	H-atom parameters constrained
*S* = 1.11	*w* = 1/[σ^2^(*F*_o_^2^) + (0.0308*P*)^2^ + 23.1984*P*] *P* = (*F*_o_^2^ + 2*F*_c_^2^)/3
3113 reflections	(Δ/σ)_max_ = 0.004
208 parameters	Δρ_max_ = 2.74 e Å^−^^3^
0 restraints	Δρ_min_ = −0.99 e Å^−^^3^

### Special details

**Table:**

Geometry. All e.s.d.'s (except the e.s.d. in the dihedral angle between two l.s. planes) are estimated using the full covariance matrix. The cell e.s.d.'s are taken into account individually in the estimation of e.s.d.'s in distances, angles and torsion angles; correlations between e.s.d.'s in cell parameters are only used when they are defined by crystal symmetry. An approximate (isotropic) treatment of cell e.s.d.'s is used for estimating e.s.d.'s involving l.s. planes.
Refinement. Refinement of *F*^2^ against ALL reflections. The weighted *R*-factor *wR* and goodness of fit *S* are based on *F*^2^, conventional *R*-factors *R* are based on *F*, with *F* set to zero for negative *F*^2^. The threshold expression of *F*^2^ > σ(*F*^2^) is used only for calculating *R*-factors(gt) *etc*. and is not relevant to the choice of reflections for refinement. *R*-factors based on *F*^2^ are statistically about twice as large as those based on *F*, and *R*- factors based on ALL data will be even larger.

### Fractional atomic coordinates and isotropic or equivalent isotropic displacement parameters (Å^2^)

**Table:**

	*x*	*y*	*z*	*U*_iso_*/*U*_eq_	
Pt1	0.625760 (7)	0.723387 (19)	0.445111 (9)	0.00847 (8)	
C5	0.6352 (2)	0.8997 (5)	0.5226 (2)	0.0123 (9)	
C12	0.6322 (2)	0.9000 (5)	0.3769 (2)	0.0119 (9)	
C15	0.5845 (2)	0.5524 (5)	0.4975 (2)	0.0118 (9)	
H15A	0.5749	0.6041	0.5353	0.014*	
C19	0.6452 (2)	0.5268 (5)	0.3793 (2)	0.0110 (9)	
H19A	0.6613	0.5682	0.3458	0.013*	
C18	0.5828 (2)	0.5402 (5)	0.3479 (2)	0.0137 (9)	
H18A	0.5628	0.5894	0.2962	0.016*	
C9	0.6352 (2)	1.1307 (5)	0.2696 (2)	0.0126 (9)	
C11	0.6864 (2)	0.9413 (5)	0.3760 (2)	0.0106 (9)	
H11A	0.7225	0.8924	0.4116	0.013*	
C8	0.6356 (2)	1.2543 (6)	0.2132 (3)	0.0172 (10)	
H8A	0.6763	1.2668	0.2210	0.026*	
H8B	0.6212	1.3575	0.2213	0.026*	
H8C	0.6096	1.2174	0.1614	0.026*	
C21	0.6671 (2)	0.3611 (5)	0.5002 (2)	0.0139 (9)	
H21A	0.6357	0.2780	0.4800	0.017*	
H21B	0.7020	0.3150	0.5446	0.017*	
C20	0.6854 (2)	0.4039 (5)	0.4380 (3)	0.0148 (9)	
H20A	0.7266	0.4459	0.4633	0.018*	
H20B	0.6854	0.3042	0.4112	0.018*	
C6	0.5846 (2)	0.9915 (5)	0.5136 (3)	0.0136 (9)	
H6A	0.5472	0.9738	0.4703	0.016*	
C7	0.5889 (2)	1.1085 (5)	0.5680 (3)	0.0162 (10)	
H7A	0.5545	1.1665	0.5601	0.019*	
C1	0.6475 (3)	1.2679 (6)	0.6911 (3)	0.0246 (12)	
H1A	0.6883	1.2740	0.7328	0.037*	
H1B	0.6205	1.2389	0.7112	0.037*	
H1C	0.6358	1.3723	0.6659	0.037*	
C13	0.5800 (2)	0.9798 (6)	0.3221 (3)	0.0153 (9)	
H13A	0.5431	0.9575	0.3207	0.018*	
C17	0.5429 (2)	0.4230 (6)	0.3636 (2)	0.0144 (9)	
H17A	0.5622	0.3163	0.3757	0.017*	
H17B	0.5044	0.4120	0.3168	0.017*	
C14	0.5813 (2)	1.0902 (5)	0.2700 (3)	0.0154 (9)	
H14A	0.5452	1.1389	0.2342	0.019*	
C22	0.6441 (2)	0.5074 (5)	0.5265 (2)	0.0133 (9)	
H22A	0.6694	0.5349	0.5812	0.016*	
C4	0.6897 (2)	0.9310 (5)	0.5893 (2)	0.0136 (9)	
H4A	0.7241	0.8714	0.5984	0.016*	
C10	0.6876 (2)	1.0531 (5)	0.3235 (2)	0.0134 (9)	
H10A	0.7244	1.0763	0.3246	0.016*	
C2	0.6439 (2)	1.1394 (6)	0.6337 (3)	0.0181 (10)	
C16	0.5305 (2)	0.4754 (6)	0.4297 (3)	0.0152 (9)	
H16A	0.4972	0.5528	0.4098	0.018*	
H16B	0.5179	0.3801	0.4478	0.018*	
C3	0.6939 (2)	1.0492 (6)	0.6427 (2)	0.0158 (9)	
H3A	0.7314	1.0683	0.6857	0.019*	

### Atomic displacement parameters (Å^2^)

**Table:**

	*U*^11^	*U*^22^	*U*^33^	*U*^12^	*U*^13^	*U*^23^
Pt1	0.01211 (11)	0.00610 (11)	0.00793 (11)	−0.00017 (6)	0.00537 (8)	−0.00012 (6)
C5	0.019 (2)	0.009 (2)	0.012 (2)	−0.0001 (18)	0.0095 (19)	0.0006 (17)
C12	0.015 (2)	0.009 (2)	0.009 (2)	0.0004 (17)	0.0042 (18)	−0.0013 (17)
C15	0.021 (2)	0.007 (2)	0.011 (2)	−0.0015 (18)	0.0101 (19)	0.0012 (16)
C19	0.021 (2)	0.004 (2)	0.011 (2)	−0.0019 (17)	0.0103 (19)	−0.0031 (16)
C18	0.022 (3)	0.009 (2)	0.009 (2)	−0.0033 (18)	0.0059 (19)	−0.0051 (16)
C9	0.023 (2)	0.007 (2)	0.0093 (19)	−0.0021 (18)	0.0085 (19)	−0.0019 (16)
C11	0.013 (2)	0.006 (2)	0.010 (2)	0.0017 (17)	0.0036 (18)	0.0008 (16)
C8	0.028 (3)	0.011 (2)	0.016 (2)	0.0040 (19)	0.014 (2)	0.0011 (18)
C21	0.018 (2)	0.010 (2)	0.013 (2)	0.0005 (18)	0.0080 (19)	0.0011 (17)
C20	0.021 (2)	0.009 (2)	0.016 (2)	0.0010 (18)	0.010 (2)	−0.0024 (17)
C6	0.018 (2)	0.007 (2)	0.016 (2)	0.0005 (17)	0.0080 (19)	0.0019 (17)
C7	0.024 (3)	0.009 (2)	0.022 (2)	0.0046 (19)	0.016 (2)	0.0041 (18)
C1	0.041 (3)	0.011 (2)	0.023 (3)	0.002 (2)	0.017 (3)	−0.0013 (19)
C13	0.016 (2)	0.016 (2)	0.015 (2)	−0.0010 (18)	0.0082 (19)	0.0001 (18)
C17	0.014 (2)	0.014 (2)	0.013 (2)	−0.0039 (18)	0.0046 (18)	−0.0045 (17)
C14	0.018 (2)	0.013 (2)	0.012 (2)	0.0049 (18)	0.0049 (19)	0.0042 (18)
C22	0.020 (2)	0.011 (2)	0.010 (2)	−0.0017 (18)	0.0079 (19)	0.0001 (17)
C4	0.017 (2)	0.011 (2)	0.014 (2)	0.0019 (18)	0.0085 (19)	0.0035 (17)
C10	0.016 (2)	0.012 (2)	0.014 (2)	−0.0029 (18)	0.0085 (19)	−0.0013 (17)
C2	0.035 (3)	0.011 (2)	0.014 (2)	0.000 (2)	0.017 (2)	0.0016 (18)
C16	0.014 (2)	0.014 (2)	0.018 (2)	−0.0009 (18)	0.0069 (19)	−0.0024 (18)
C3	0.021 (2)	0.014 (2)	0.009 (2)	−0.0057 (19)	0.0044 (19)	−0.0007 (18)

### Geometric parameters (Å, °)

**Table:**

Pt1—C5	2.028 (4)	C21—C20	1.538 (6)
Pt1—C12	2.028 (4)	C21—H21A	0.9700
Pt1—C15	2.256 (4)	C21—H21B	0.9700
Pt1—C19	2.257 (4)	C20—H20A	0.9700
Pt1—C18	2.258 (4)	C20—H20B	0.9700
Pt1—C22	2.279 (4)	C6—C7	1.399 (6)
C5—C4	1.394 (6)	C6—H6A	0.9300
C5—C6	1.409 (6)	C7—C2	1.392 (7)
C12—C13	1.399 (6)	C7—H7A	0.9300
C12—C11	1.405 (6)	C1—C2	1.514 (7)
C15—C22	1.369 (7)	C1—H1A	0.9600
C15—C16	1.511 (6)	C1—H1B	0.9600
C15—H15A	0.9800	C1—H1C	0.9600
C19—C18	1.383 (6)	C13—C14	1.376 (6)
C19—C20	1.500 (6)	C13—H13A	0.9300
C19—H19A	0.9800	C17—C16	1.535 (6)
C18—C17	1.517 (6)	C17—H17A	0.9700
C18—H18A	0.9800	C17—H17B	0.9700
C9—C10	1.387 (6)	C14—H14A	0.9300
C9—C14	1.393 (7)	C22—H22A	0.9800
C9—C8	1.504 (6)	C4—C3	1.393 (6)
C11—C10	1.390 (6)	C4—H4A	0.9300
C11—H11A	0.9300	C10—H10A	0.9300
C8—H8A	0.9600	C2—C3	1.389 (7)
C8—H8B	0.9600	C16—H16A	0.9700
C8—H8C	0.9600	C16—H16B	0.9700
C21—C22	1.521 (6)	C3—H3A	0.9300
			
C5—Pt1—C12	88.54 (18)	H21A—C21—H21B	107.7
C5—Pt1—C15	90.66 (17)	C19—C20—C21	114.9 (4)
C12—Pt1—C15	160.11 (17)	C19—C20—H20A	108.5
C5—Pt1—C19	163.18 (18)	C21—C20—H20A	108.5
C12—Pt1—C19	91.14 (17)	C19—C20—H20B	108.5
C15—Pt1—C19	95.26 (16)	C21—C20—H20B	108.5
C5—Pt1—C18	161.08 (18)	H20A—C20—H20B	107.5
C12—Pt1—C18	93.84 (17)	C7—C6—C5	122.0 (4)
C15—Pt1—C18	80.76 (16)	C7—C6—H6A	119.0
C19—Pt1—C18	35.68 (16)	C5—C6—H6A	119.0
C5—Pt1—C22	96.28 (17)	C2—C7—C6	121.1 (4)
C12—Pt1—C22	164.40 (17)	C2—C7—H7A	119.4
C15—Pt1—C22	35.13 (16)	C6—C7—H7A	119.4
C19—Pt1—C22	79.97 (16)	C2—C1—H1A	109.5
C18—Pt1—C22	86.41 (16)	C2—C1—H1B	109.5
C4—C5—C6	116.0 (4)	H1A—C1—H1B	109.5
C4—C5—Pt1	123.3 (3)	C2—C1—H1C	109.5
C6—C5—Pt1	120.6 (3)	H1A—C1—H1C	109.5
C13—C12—C11	115.5 (4)	H1B—C1—H1C	109.5
C13—C12—Pt1	120.2 (3)	C14—C13—C12	122.4 (4)
C11—C12—Pt1	124.1 (3)	C14—C13—H13A	118.8
C22—C15—C16	126.5 (4)	C12—C13—H13A	118.8
C22—C15—Pt1	73.4 (3)	C18—C17—C16	114.5 (4)
C16—C15—Pt1	105.5 (3)	C18—C17—H17A	108.6
C22—C15—H15A	114.2	C16—C17—H17A	108.6
C16—C15—H15A	114.2	C18—C17—H17B	108.6
Pt1—C15—H15A	114.2	C16—C17—H17B	108.6
C18—C19—C20	126.7 (4)	H17A—C17—H17B	107.6
C18—C19—Pt1	72.2 (2)	C13—C14—C9	121.7 (4)
C20—C19—Pt1	106.5 (3)	C13—C14—H14A	119.2
C18—C19—H19A	114.2	C9—C14—H14A	119.2
C20—C19—H19A	114.2	C15—C22—C21	125.7 (4)
Pt1—C19—H19A	114.2	C15—C22—Pt1	71.5 (3)
C19—C18—C17	124.9 (4)	C21—C22—Pt1	110.6 (3)
C19—C18—Pt1	72.1 (2)	C15—C22—H22A	113.7
C17—C18—Pt1	109.8 (3)	C21—C22—H22A	113.7
C19—C18—H18A	114.1	Pt1—C22—H22A	113.7
C17—C18—H18A	114.1	C3—C4—C5	121.8 (4)
Pt1—C18—H18A	114.1	C3—C4—H4A	119.1
C10—C9—C14	117.0 (4)	C5—C4—H4A	119.1
C10—C9—C8	122.3 (4)	C9—C10—C11	121.4 (4)
C14—C9—C8	120.7 (4)	C9—C10—H10A	119.3
C10—C11—C12	122.0 (4)	C11—C10—H10A	119.3
C10—C11—H11A	119.0	C3—C2—C7	117.1 (4)
C12—C11—H11A	119.0	C3—C2—C1	122.9 (5)
C9—C8—H8A	109.5	C7—C2—C1	120.0 (5)
C9—C8—H8B	109.5	C15—C16—C17	114.0 (4)
H8A—C8—H8B	109.5	C15—C16—H16A	108.7
C9—C8—H8C	109.5	C17—C16—H16A	108.7
H8A—C8—H8C	109.5	C15—C16—H16B	108.7
H8B—C8—H8C	109.5	C17—C16—H16B	108.7
C22—C21—C20	113.4 (4)	H16A—C16—H16B	107.6
C22—C21—H21A	108.9	C2—C3—C4	122.1 (4)
C20—C21—H21A	108.9	C2—C3—H3A	119.0
C22—C21—H21B	108.9	C4—C3—H3A	119.0
C20—C21—H21B	108.9		
			
C12—Pt1—C5—C4	−100.7 (4)	C12—Pt1—C18—C17	−151.9 (3)
C15—Pt1—C5—C4	99.1 (4)	C15—Pt1—C18—C17	8.8 (3)
C19—Pt1—C5—C4	−11.6 (8)	C19—Pt1—C18—C17	121.5 (4)
C18—Pt1—C5—C4	161.7 (4)	C22—Pt1—C18—C17	43.7 (3)
C22—Pt1—C5—C4	64.4 (4)	C13—C12—C11—C10	−0.6 (6)
C12—Pt1—C5—C6	83.4 (4)	Pt1—C12—C11—C10	174.2 (3)
C15—Pt1—C5—C6	−76.7 (4)	C18—C19—C20—C21	−37.3 (6)
C19—Pt1—C5—C6	172.5 (4)	Pt1—C19—C20—C21	42.5 (4)
C18—Pt1—C5—C6	−14.2 (7)	C22—C21—C20—C19	−38.0 (5)
C22—Pt1—C5—C6	−111.5 (4)	C4—C5—C6—C7	0.7 (6)
C5—Pt1—C12—C13	−89.5 (4)	Pt1—C5—C6—C7	176.9 (3)
C15—Pt1—C12—C13	−1.6 (7)	C5—C6—C7—C2	0.1 (7)
C19—Pt1—C12—C13	107.3 (4)	C11—C12—C13—C14	0.7 (6)
C18—Pt1—C12—C13	71.7 (4)	Pt1—C12—C13—C14	−174.3 (3)
C22—Pt1—C12—C13	162.1 (5)	C19—C18—C17—C16	93.6 (5)
C5—Pt1—C12—C11	95.9 (4)	Pt1—C18—C17—C16	12.0 (5)
C15—Pt1—C12—C11	−176.2 (4)	C12—C13—C14—C9	−0.9 (7)
C19—Pt1—C12—C11	−67.3 (4)	C10—C9—C14—C13	0.8 (6)
C18—Pt1—C12—C11	−102.9 (4)	C8—C9—C14—C13	−178.7 (4)
C22—Pt1—C12—C11	−12.5 (8)	C16—C15—C22—C21	−5.3 (7)
C5—Pt1—C15—C22	−100.0 (3)	Pt1—C15—C22—C21	−102.5 (4)
C12—Pt1—C15—C22	172.5 (4)	C16—C15—C22—Pt1	97.2 (4)
C19—Pt1—C15—C22	64.2 (3)	C20—C21—C22—C15	94.0 (5)
C18—Pt1—C15—C22	96.9 (3)	C20—C21—C22—Pt1	12.4 (5)
C5—Pt1—C15—C16	135.8 (3)	C5—Pt1—C22—C15	82.2 (3)
C12—Pt1—C15—C16	48.3 (6)	C12—Pt1—C22—C15	−170.5 (5)
C19—Pt1—C15—C16	−60.0 (3)	C19—Pt1—C22—C15	−114.4 (3)
C18—Pt1—C15—C16	−27.3 (3)	C18—Pt1—C22—C15	−79.0 (3)
C22—Pt1—C15—C16	−124.2 (4)	C5—Pt1—C22—C21	−155.7 (3)
C5—Pt1—C19—C18	176.3 (5)	C12—Pt1—C22—C21	−48.4 (7)
C12—Pt1—C19—C18	−95.0 (3)	C15—Pt1—C22—C21	122.1 (4)
C15—Pt1—C19—C18	66.1 (3)	C19—Pt1—C22—C21	7.7 (3)
C22—Pt1—C19—C18	97.9 (3)	C18—Pt1—C22—C21	43.1 (3)
C5—Pt1—C19—C20	52.3 (7)	C6—C5—C4—C3	−1.6 (6)
C12—Pt1—C19—C20	141.0 (3)	Pt1—C5—C4—C3	−177.6 (3)
C15—Pt1—C19—C20	−57.9 (3)	C14—C9—C10—C11	−0.7 (6)
C18—Pt1—C19—C20	−124.0 (4)	C8—C9—C10—C11	178.9 (4)
C22—Pt1—C19—C20	−26.1 (3)	C12—C11—C10—C9	0.6 (7)
C20—C19—C18—C17	−4.5 (7)	C6—C7—C2—C3	−0.1 (6)
Pt1—C19—C18—C17	−102.1 (4)	C6—C7—C2—C1	179.4 (4)
C20—C19—C18—Pt1	97.6 (4)	C22—C15—C16—C17	−37.5 (6)
C5—Pt1—C18—C19	−176.7 (4)	Pt1—C15—C16—C17	43.0 (4)
C12—Pt1—C18—C19	86.6 (3)	C18—C17—C16—C15	−38.4 (5)
C15—Pt1—C18—C19	−112.7 (3)	C7—C2—C3—C4	−0.8 (7)
C22—Pt1—C18—C19	−77.8 (3)	C1—C2—C3—C4	179.7 (4)
C5—Pt1—C18—C17	−55.2 (6)	C5—C4—C3—C2	1.7 (7)

### Hydrogen-bond geometry (Å, °)

**Table:**

Cg1 and Cg2 are the centroids of the C9–C14 and C2–C7 rings, respectively.

**Table:**

*D*—H···*A*	*D*—H	H···*A*	*D*···*A*	*D*—H···*A*
C1—H1B···Cg1^i^	0.96	2.93	3.615 (4)	129
C20—H20B···Cg1^ii^	0.96	2.85	3.749 (5)	155
C21—H21A···Cg2^ii^	0.97	2.83	3.411 (4)	119
C8—H8C···Cg2^iii^	0.96	2.85	3.509 (2)	126

Symmetry codes: (i) *x*, −*y*+2, *z*+1/2; (ii) *x*, *y*−1, *z*; (iii) *x*, −*y*+2, *z*−1/2.
